# An Evaluation of Morphometric Characteristics of Honey Bee (*Apis cerana*) Populations in the Qinghai–Tibet Plateau in China

**DOI:** 10.3390/life15020255

**Published:** 2025-02-07

**Authors:** Xinru Zhang, Jian Lu, Xinying Qu, Xiao Chen

**Affiliations:** 1 State Key Laboratory of Resource Insects, Institute of Apicultural Research, Chinese Academy of Agricultural Sciences, Beijing 100193, China; 202330112016@bua.edu.cn (X.Z.); 82101235489@caas.cn (X.Q.); 2 College of Bioscience and Resource Environment, Beijing University of Agriculture, Beijing 102206, China; 3 National Animal Husbandry Station, Beijing 100125, China; lujian34@163.com

**Keywords:** *Apis cerana*, Qinghai–Tibetan Plateau, diversity, morphometric, honey bee conservation

## Abstract

*Apis cerana*, a native type of honey bee in China, adapts well to the Qinghai–Tibet Plateau (QTP) environments with high altitude, cold, low oxygen, and strong radiation. In this study, we detected 40 morphological characteristics from 100 colonies in 49 regions. We not only evaluated the morphometric characteristics of honey bee populations in the QTP but also found that the pigmentation of labrum and tergite 2 in *A. cerana* is significantly different from that in *Apis mellifera*. Moreover, most morphological characteristics were correlated with environmental factors. Tibet and Qinghai could be distinctly separated. The cluster analysis indicated that Xunhua and Danba were far apart and formed a single cluster. Honey bees from Danba and Linzhichayu were correctly judged into corresponding populations. There was large morphometric diversity within the selected sampling areas of the Sichuan, Yunnan, and Gansu populations. Overall, our findings offer insights into the conservation and sustainable utilization of *A. cerana* in the QTP.

## 1. Introduction

*Apis cerana* from China is a species and type of native honey bee that may have originated in the southern region of Yunnan Province in China and expanded from there into the peripheral valleys of the Qinghai–Tibetan Plateau (QTP) [1]. In the long-term evolution process, *A. cerana* has adapted to the local climate and honey source conditions and become an important component of the natural ecosystem in China [2]. *Apis cerana* is also the main managed bee in China, aside from *Apis mellifera* [3]. In 2008, there were 2.8 million *A. cerana* colonies in China, and 5.16 million in 2014 [4]. By the end of 2016, the number of *A. cerana* colonies was close to 6 million [5]. This reflects the increasingly important role of *A. cerana* in modern agriculture. Protecting and utilizing *A. cerana* has become more important.

*Apis cerana* inhabiting different regions often exhibit different phenotypes and behavioral adaptations [6]. Climate types, geographic locations, and food resources are all related to the unique geographic phenotypes of *A. cerana* [7,8]. The change in the ecological environment, especially the change in altitude and latitude, is the main cause of morphological variation in *A. cerana* [9]. The Qinghai–Tibetan Plateau (QTP) is located in southwestern China, west of the Hengduan Mountains, north of the Himalayas, and south of the Kunlun, Altyn, and Qilian Mountains [10]. The QTP region includes Qinghai Province, the Tibet Autonomous Region, western Sichuan, southwestern Gansu, and the southern edge of Xinjiang, with an average altitude of 4000 m [11]. Given the complex geographical environment and diverse ecosystem conditions, honey bees have abundant genetic resources [12,13]. Limited by the availability of appropriate nesting conditions, low temperatures, and insufficient nectar sources [14], *A. cerana* was mainly observed below 3500 m [15]; the eastern and southern parts of the QTP could be suitable for the residence of *A. cerana* [16]. This area is also considered an important biodiversity hotspot and a priority area that requires pollinator protection [17,18]. Studying the characteristics and genetic diversity of *A. cerana* is crucial not only for protecting honey bees but also for the unique environment of the QTP [16]. Phenotypic plasticity, a universal property of living things, is the property of organisms to produce distinct phenotypes in response to environmental variation [19]. To some extent, phenotypic variations in response to environmental change reflect an underlying genetic polymorphism [20,21,22,23]. Many researchers use morphological methods as the main means to analyze the genetic diversity of honey bees [24,25]. Traditionally, the intraspecific taxonomy of honey bees has been based on morphology [26]. In 1988, Ruttner established a large database with 38 standard characteristics. The database serves as an international reference for the biogeography of honey bees [27]. More comprehensive morphometric methods and complex statistical analyses have been widely used for the scientific discrimination of honey bee populations and to make sure that the breeding populations in the national breeding program are pure, which has successfully identified many new honey bee subspecies in different countries in the world [28]. Then, in 2013, Meixner et al. gave an overview of standard methods currently available and used for distinguishing subspecies and ecotypes of *A. mellifera*, which can be utilized to verify the genetic origin of colonies for breeding purposes [29]. Therefore, studying the morphometric characteristics of honey bees in the QTP is of great significance.

Previous research on population structure and classification of *A. cerana* covered parts of QTP samples, like northern areas of Yunnan [30], southern Gansu [24] and southern Tibet [31]. In 2003, Tan et al. used morphometrical methods and found that bees from the northern high-altitude areas of Yunnan Province were clearly larger and darker and showed similarities with samples from Nepal [30]. In 2010, Radloff revealed that bees from southern Gansu were of morphocluster I, and some bees in south Tibet and Nepal belonged to morphocluster II, which was known as the “Himalayan cerana” [24]. In 2015, Tan et al. analyzed the Himalayan region and areas in southwestern China based on mtDNA diversity and detected 14 haplotypes but were unable to resolve any relationships among the haplotypes, reflective of extensive reticulation and lack of population differentiation across the region [31]. These studies indicated that the genetic diversity of *A. cerana* in the QTP is abundant. However, these studies did not fully cover and explore the diversity of *A. cerana* in all regions of the QTP. They either only included two to four commonly known sampling sites in the QTP, or the sampling sites were skewed toward a certain region, such as Sichuan Province and the Qinghai–Tibet Plateau–Valley region, leaving many regions in the QTP unexplored [15,32,33,34]. In addition, the detected morphological characteristics were not comprehensive and could not fully reflect the characteristics of *A. cerana* in the QTP.

In order to overcome the lack of diversity of bee samples in the QTP and fill in the research gaps, we collected samples from 100 colonies in 49 regions across the QTP and utilized the morphological analysis method to reveal the characteristics of bee genetic resources. It is crucial that we analyze and classify the characteristics of *A. cerana* to evaluate genetic diversity in the QTP. Here, we found four types of labrum pigmentation and scored them on the basis of the standard method of *A. mellifera*, which enhanced the classification criteria of *A. cerana*. Simultaneously, we chose collection sites at lower altitudes to explore the influence of geographical coordinate factors on the phenotypic variations in *A. cerana*. This study provides a useful reference for improving *A. cerana* conservation and better utilization in the QTP.

## 2. Materials and Methods

### 2.1. Description of the Area of Study

**Figure 1:**
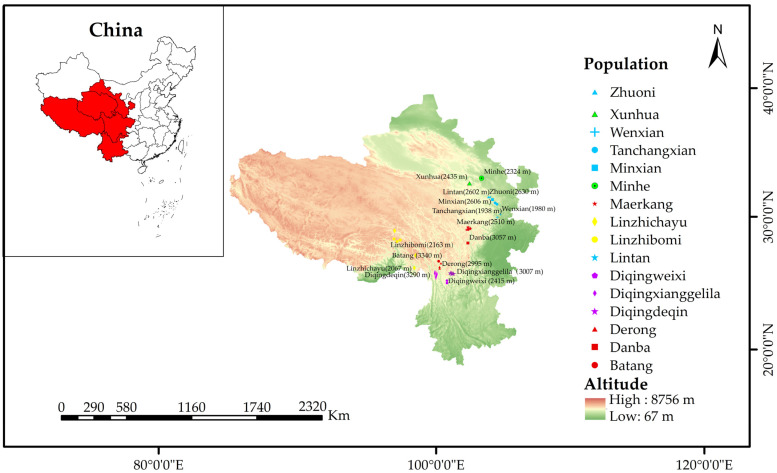
Locations of *A. cerana* sampling sites.

The area of study (Figure 1) is located approximately between 99°22′39.8″ E and 104°55′19.8″ E and 27°08′05.4″ N and 30°16′06.6″ N, with an altitude ranging from 1581 m to 3393 m. It covers parts of Sichuan Province, Yunnan Province, Gansu Province, Qinghai Province, and Tibet. This is the QTP region where *A. cerana* is distributed in China. The terrain is mainly mountainous and consists of plateaus. The climate is temperate with warm temperate zones. As a control, the non-plateau sample site of Shandong (eastern China) is located at 35°45′11″ N and 107°58′60″ E, and the altitude is 540 m. A visualization of the important geographical features of the area is given below.

### 2.2. Collection of Honey Bees

Samples of honey bee workers were collected from 100 colonies in 16 counties in the 5 provinces (Figure 1). They were collected from unmanaged traditional or top-bar hives populated by wild swarms. In some cases, some hives were not easily opened for a variety of reasons (such as traditional hives, natural comb, defensive bees, etc.), so sampling from the flight entrance sufficed. We randomly selected a basket of bees from the hives, preferably from the brood area (which we visually observed to have a strong, fluffy appearance) and a uniform color on the comb. We then placed them into a sealed bag and closed it until the bees calmed down or were exposed to the sun for a while. When most of the bees were dead, we transferred them into a 50 mL centrifuge tube and preserved them in 75% ethanol. The collected samples and the generated morphometric data were stored at the Institute of Apicultural Research, Chinese Academy of Agricultural Sciences, Beijing.

### 2.3. Sample Preparation and Morphometric Measurements

Sample preparation and morphometric measurements of 40 characteristics were taken from 10 bees from each colony, following the method described by Ruttner and Meixner (Table S1) [27,29]. Hair measurements were taken under a ZEISS dissecting microscope (Stemi 508) (Oberkochen, Germany) fitted with an eyepiece graticule at a 32× magnification. Wings, legs, and sternite measurements were taken using LEICA DMS 300 with a CCD camera (Wetzlar, Germany) connected to a desktop computer.

### 2.4. Statistical Analyses

The mean, standard deviation, and coefficient of variation in each of the 40 morphometric characteristics were calculated for every colony. The data were subjected to a one-way analysis of variance (ANOVA) to compare results from different counties. Tukey’s HSD tests were used to detect significant differences between the counties on the means of the 40 characteristics. The Bonferroni method was used for multiple comparisons to correct the *p*-value. Prior to analysis, the data were assessed for linearity, normal distribution, and the presence of outliers. Spearman’s product–moment correlation analyses of morphologic features and morphology features between latitude, longitude, and altitude were carried out using OriginPro^®^2024b. Interpretation of the magnitude of Spearman’s correlation coefficient, r, was based on Cohen [35], that is, 0.1 < |r| < 0.3: low correlation; 0.3 < |r| < 0.5: medium correlation; |r| > 0.5: high correlation. ** *p*-value ≤ 0.01: correlation is significant at the 0.01 level. * *p*-value ≤ 0.05: correlation is significant at the 0.05 level. *p*-value < 0.05: significant difference; *p*-value <0.01: extremely significant difference. Using colony means of 40 morphometric characteristics, a PCA was run to visually detect any possible clusters from the plot. The suitability of PCA was assessed prior to the analysis using the scaled variables, Kaiser–Meyer–Olkin (KMO) measure of sampling adequacy, and Bartlett’s test of sphericity. The KMO measure is used as an index of whether the variables have linear relationships. Its value can range from 0 to 1, with values above 0.6 suggested as a minimum requirement for sampling adequacy. Bartlett’s test of sphericity tests the null hypothesis, which states that there can be no correlations between any of the variables; therefore, the variables cannot be reduced to a smaller number of principal components. For a PCA to be feasible, the null hypothesis must be rejected. The result of Bartlett’s test was used to make this decision. “Eigenvalues greater than 1” serve as the standard for determining the number of principal components. To study the morphological differentiation of honey bees under different habitats, stepwise discriminant was performed using the 40 morphological characteristics, and then cluster analysis was carried out using the means discriminant functions scores of each individual from 16 sampling counties to calculate the Square Euclidean Distance. Population differentiation was determined using cluster analysis. Then, stepwise discriminant analysis (DA) was run in order to confirm the groups predicted by cluster analysis and to determine the discriminant characteristics. Statistical analyses were carried out using IBM^®^ SPSS^®^ Statistics Version 27.0, and the graphs were visualized with OriginPro^®^ 2024b.

## 3. Results

### 3.1. Pigmentation of Labrum, Scutellum, and Tergite

**Figure 2:**
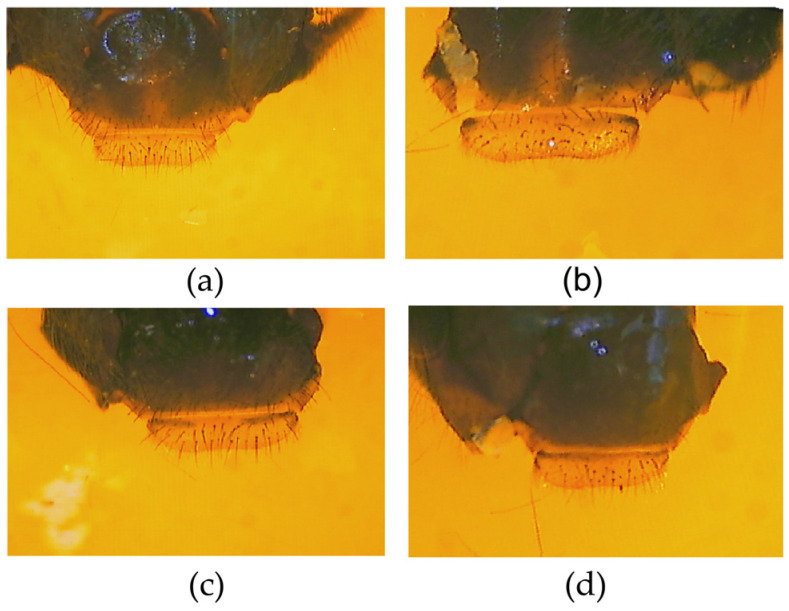
The pigmentation of the labrum in *A. cerana*: (**a**) 60, the labrum is completely yellow; (**b**) 55, little black patches on both ends of the labrum; (**c**) 55, the black gradually lengthens to form a line, but the two ends of the line are not connected; (**d**) 53, the black extension at either end of the labrum that forms a long line.

**Figure 3:**
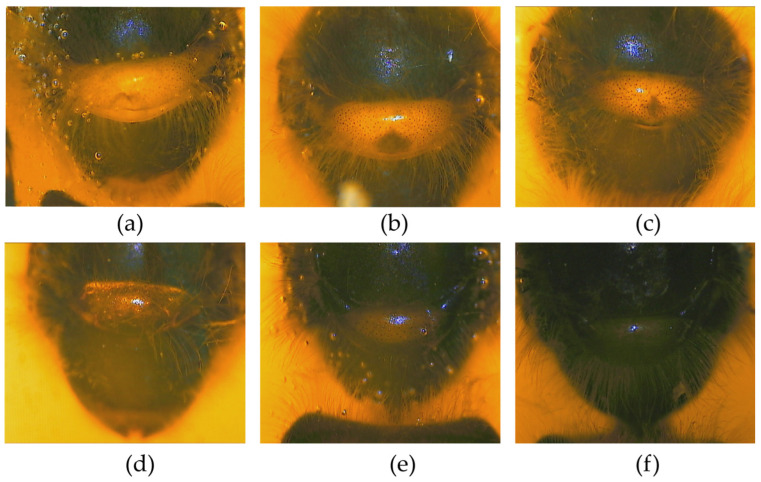
The pigmentation of scutellum cupola, K and B in *A. cerana*. (**a**) Scutellum cupola, K and B totally yellow; (**b**) little dark in scutellum cupola, K yellow and B dark; (**c**) little dark in scutellum cupola, little yellow in K and B dark; (**d**) a smaller bright area in scutellum cupola, K dark and B dark; (**e**) scutellum cupola brown, K dark and B dark; (**f**) scutellum cupola, K and B totally dark.

**Figure 4:**
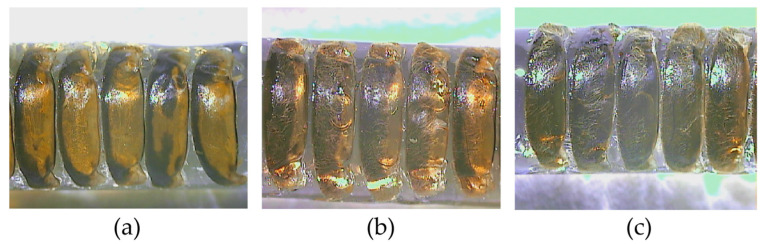
The pigmentation of tergite 2 in *A. cerana*. (**a**) Tergite 2 with light pigmentation. (**b**) Tergite 2 with medium dark pigmentation. (**c**) Tergite 2 with dark pigmentation.

The pigmentation of labrum (*PLAB*) and pigmentation of tergite 2 (*PT2*) is significantly different from that in *A. mellifera* (Figures S1 and S2). Four types of *PLAB* were found and scored as follows (Figure 2). The first type of *PLAB* is completely yellow and scored 60. The second type comprises little black patches on both ends of the labrum, scoring 55. The third type is that the black gradually lengthens to form a line, but the two ends of the line are not connected, scoring 55. The fourth type is the black extension at either end of the labrum which forms a long line, scoring 53. The pigmentation of scutellum cupola, B and K in *A. cerana* are similar to that of *A. mellifera*. Six types were found (Figure 3). The pigmentation of tergite 3 (*PT3*) and tergite 4 (*PT4*) is similar to that of *A. mellifera*. However, *PT2* is different from that in *A. mellifera* (Figure 4). The distribution of the light part and the dark part does not align well with the standard method of morphological identification of *A. mellifera* (Figure S2). But the distribution of the light part and dark part shows a changing pattern. So *PT2* was scored on a scale of 0~9 from black to light.

### 3.2. Body Size Characteristics

The ANOVA results showed that there were significant differences (*p* < 0.05) between the measured morphological characteristics and the different counties (Table S2). Hair length (*HLT5*) ranged from 0.49 mm to 1.17 mm, with bees from Gansu Wenxian recording the longest lengths than other counties. The forewing length (*FWL*) ranged from 7.94 mm to 9.08 mm. The *FWL* of bees collected from Sichuan Batang, where the altitude is highest, was significantly larger than the *FWL* of those from Linzhichayu (*p* < 0.001). The forewing (*FWW*) width ranged from 2.87 mm to 3.25 mm. The *FWW* of the bees in Lintan was significantly larger than Linzhhichayu (*p* < 0.001). The shortest leg, including tibia (*TIB*), basitatrus length (*TAL*), and basitatrus width (*TAW*) in Tibet Linzhichayu, was 7.18 mm. In the country, the longest leg was 7.88 mm from bees in Yunnan Diqingdeqin, where the altitude is over 3000 m. The longest length of tergite 3 (*T3*) from bees in Sichuan Derong was significantly larger than in Xunhua (*p* = 0.002). However, there was no significant difference in the length of tergite 4 (*T4*). Tergite 3 and 4, longitudinal (*T3+4*) of bees in Derong, is significantly larger than Tanchangxian (*p* = 0.032), where the altitude is lowest in the QTP. Compared with the QTP samples, the body size of Shandong varied to a smaller degree. The length of *LEG*, including *TIB*, *TAL*, and *TAW*, and the forewing features, including *FWL* and *FWW*, was significantly shorter than other counties, except for Tibet Linzhichayu and Linzhibomi. The results of these characteristics showed that the phenotypic changes in bees are associated with the geographical conditions of their habitat. 

The coefficient of variation (*CV*) is an index used to measure the genetic diversity of bees [36,37]. The *CV* of *HLT5* ranges from 0.0314 to 0.2532; the maximum value is Minxian, and the minimum is from bees in Xunhua (Table S3). The *CV* of *FWL* ranges from 0.0039 to 0.0408; the maximum value is from Zhuoni, and the minimum is from Minxian. The *CV* of *TIB* ranges from 0.0060 to 0.0250; the maximum value is from Xunhua, and the minimum is from Minxian. The *CV* of *LEG* ranges from 0.0072 to 0.0204. Minhe is the maximum, and Danba is the minimum. The *CV* of *T3* ranges from 0.0079 to 0.0481, Maerkang is the maximum, and Xunhua is the minimum. The *CV* of *T4* ranges from 0.0026 to 0.0552. Linzhibomi is the maximum, and Danba is the minimum. The *CV* of *T3+4* ranges from 0.0044 to 0.2513, Linzhichayu is the maximum, and Tanchangxian is the minimum. By contrast, the *CV* of Shandong only *LEG* and *T4* are larger than the QTP. Compared with the non-plateau areas, the morphological characteristics of the bees in the QTP have more abundant variation. The results indicated great potential for improving and protecting the honey bee resources in the QTP.

### 3.3. Correlation Coefficient of Morphology Features

**Figure 5:**
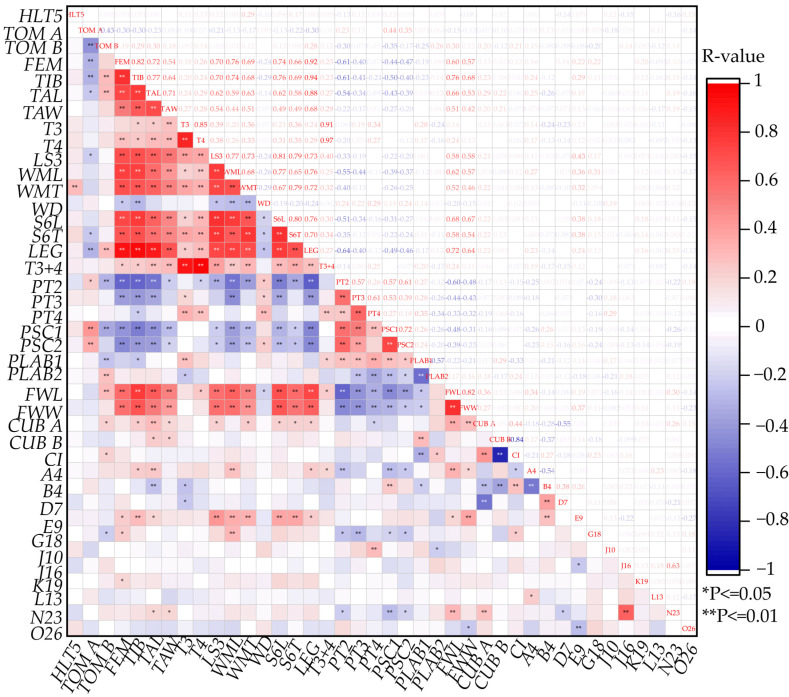
Heatmap of Spearman correlation between 40 morphology features. R-value varies from −1 to 1. Blue, negative correlation; Red, positive correlation. ** *p*-value ≤ 0.01: correlation is significant at the 0.01 level. * *p*-value ≤ 0.05: correlation is significant at the 0.05 level. Note: *HLT5*, length of cover hair on tergite 5; *TOM A*, width of tomentum on tergite 4; *TOM B*, width of stripe posterior of tomentum. *FEM*, femur; *TIB*, tibia; *TAL*, basitarsus length; *TAW*, basitarsus width; *T3*, tergite 3, longitudinal; *T4*, tergite 4, longitudinal; *LS3*, Sternite 3, longitudinal; *WML*, wax mirror of sternite 3 longitudinal. *WMT*, wax mirror of sternite 3, transversal; *WD*, distance between wax mirrors st. 3; *S6L*, sternite 6, longitudinal; *S6T*, sternite 6, transversal; *LEG*, length of hind leg; *T3+4*, tergite 3 and 4, longitudinal; *PT2*, pigmentation of tergite 2; *PT3*, pigmentation of tergite 3; *PT4*, pigmentation of tergite 4; *PSC1*, pigmentation of scutellum, cupola; *PSC2*, pigmentation of scutellum, B and K; *PLAB1*, pigmentation of labrum 1; *PLAB2*, pigmentation of labrum 2; *FWL*, forewing length; *FWW*, forewing width; *CUB A*, cubital vein, distance a; *CUB B*, cubital vein, distance b; *CI*, cubital index; *A4*, wing angle A4; *B4*: wing angle B4; *D7*, wing angle D7; *E9*, wing angle E9; *G18*, wing angle G18; *J10*, wing angle J10; *J16*, wing angle J16; *K19*, wing angle K19; *L13*, wing angle L13; *N23*, wing angle N23; *O26*, wing angle O26.

The correlation analysis showed that *HLT5* had a low correlation with other morphology features (Figure 5). The features related to the leg, such as the femur (*FEM*), *TIB*, *TAL*, and *TAW*, are highly correlated with each other, as well as the wax mirror of sternite 3 longitudinal (*WML*), wax mirror of sternite 3 transversal (*WMT*), *FWL*, *FWW*, sternite 3 longitudinal (*LS3*). The body size feature, such as *T3* and *T4*, were moderately correlated with *FEM*, *TIB*, *TAL*, *TAW*, and wing angle A4 (*A4*), to some extent, which can be seen as an index to measure the individual size of bees and reflect the size of worker honey sac. The features related to pigmentation, such as *PT2*, were highly correlated with *FEM*, *TIB*, *TAL*, *TAW*, *LEG*, *WML*, pigmentation of labrum 1 (*PLAB1*), and pigmentation of labrum 2 (*PLAB2*). The features related to the wing, such as *FWL* and *FWW*, were both highly correlated with *FEM*, *TIB*, *TAL*, *LS3*, *WML*, *WMT*, *LEG*, and *PT2*. Cubital index (*CI*) was only moderately correlated with *PLAB1* and wing angle B4 (*B4*), indicating a relatively independent feature in distinguishing honey bees. *B4* was moderately correlated with *CUBA* and *A4*. Wing angle E9 (*E9*) was moderately correlated with *T3*, *WML*, *WMT*, and *FWW*.

### 3.4. Principal Components Analysis of Morphology Features

**Figure 6:**
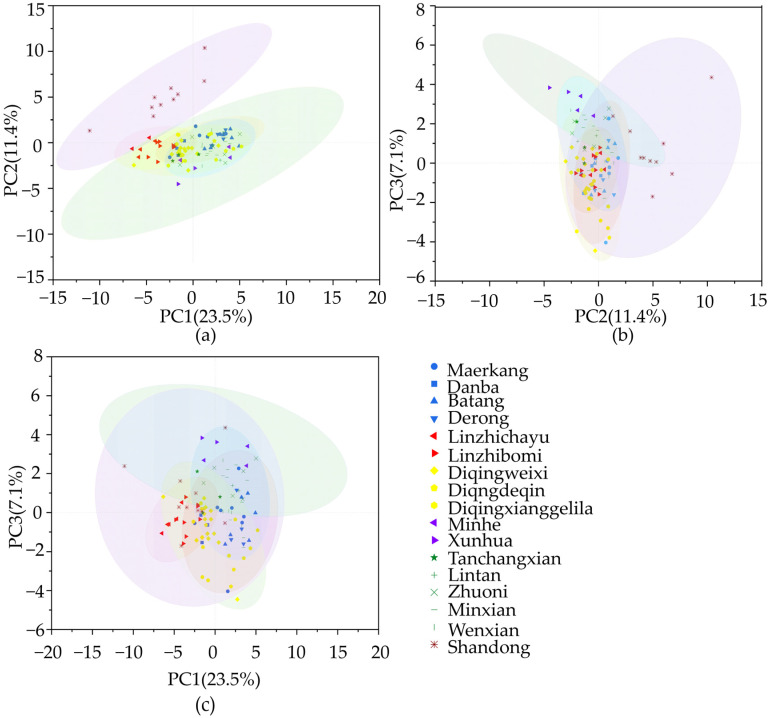
Principal component analysis plot of *A. cerana* populations based on colony means of 40 morphology features measured, with confidence ellipses at 95%. Each symbol represents one population, and different populations have different shapes. (**a**) PC1 vs. PC2; (**b**) PC2 vs. PC3; (**c**) PC1 vs. PC3.

Morphology feature values from 16 counties in 5 provinces in the QTP and the control region were performed using principal components analysis. The first principal component (PC1) accounted for 23.490% of the total variance. The second and third principal components (PC2 and PC3) accounted for 11.380% and 7.093% of the total variance, respectively. The reason for the low percentage of the total variance may be that conventional morphometry mixes information about pigmentation and size as well as shape, which makes it difficult to extract the key characteristics. The samples from Shandong Province can be separated from QTP samples. The Tibetan samples occupied the negative axis of PC1 and the center of PC2, and the Qinghai samples occupied the center of PC1 and the negative axis of PC2. Yunnan samples occupied the center of the positive axis of PC1 and the negative axis of PC2. In addition, Sichuan and Gansu samples occupied the center and positive axis of PC1 and PC2 (Figure 6). Although the samples from Tibet and Qinghai can be completely distinguished, there is an overlap with some samples from Sichuan, Gansu, and Yunnan, showing abundant morphological diversity. Further research revealed that factor 1 related to the characteristics of leg and sternite, e.g., *FEM*, *TIB*, *TAL*, *TAW*, *LS3*, *WML*, *WMT*, *FWL*, *FWW*, *PT2*, *PT3* and scutellum cupola, B and K (*PSC2*). Factor 2 is related to the characteristics of tergite, e.g., T3, *T4*, and *PT4*. Factor 3 is related to cubital vein distance b (*CUB B*) and *B4*.

### 3.5. Correlation Analysis of Morphology Features and Geographical Features

**Figure 7:**
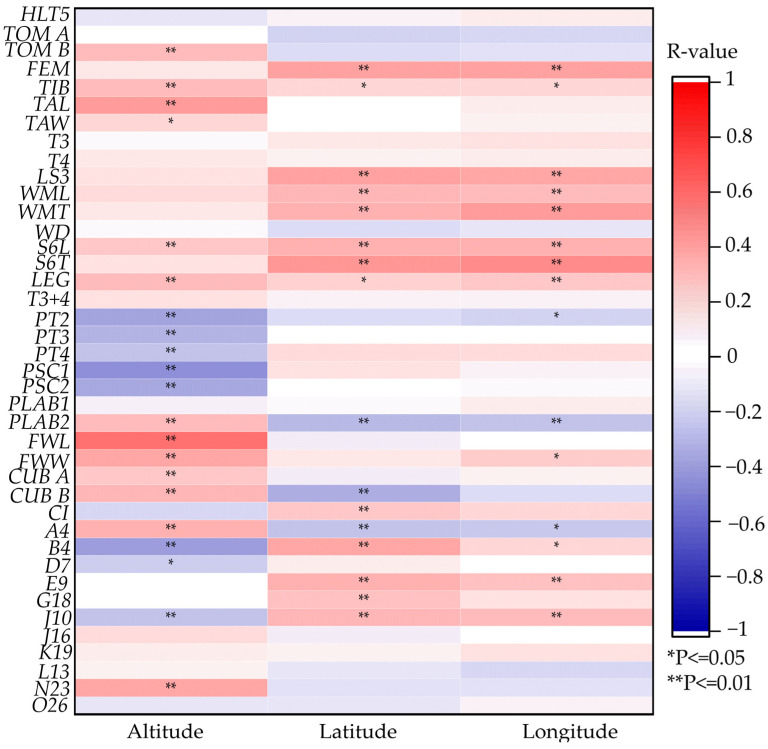
Heatmap of Spearman correlation between 40 morphology features and altitude, longitude, and latitude. R-value varies from −1 to 1. Blue, negative correlation; Red, positive correlation. ** *p*-value ≤ 0.01: correlation is significant at the 0.01 level. * *p*-value ≤ 0.05: correlation is significant at the 0.05 level. Note: *HLT5*, length of cover hair on tergite 5; *TOM A*, width of tomentum on tergite 4; *TOM B*, width of stripe posterior of tomentum. *FEM*, femur; *TIB*, tibia; *TAL*, basitarsus length; *TAW*, basitarsus width; *T3*, tergite 3, longitudinal; *T4*, tergite 4, longitudinal; *LS3*, Sternite 3, longitudinal; *WML*, wax mirror of sternite 3 longitudinal. *WMT*, wax mirror of sternite 3, transversal; *WD*, distance between wax mirrors st. 3; *S6L*, sternite 6, longitudinal; *S6T*, sternite 6, transversal; *LEG*, length of hind leg; *T3+4*, tergite 3 and 4, longitudinal; *PT2*, pigmentation of tergite 2; *PT3*, pigmentation of tergite 3; *PT4*, pigmentation of tergite 4; *PSC1*, pigmentation of scutellum, cupola; *PSC2*, pigmentation of scutellum, B and K; *PLAB1*, pigmentation of labrum 1; *PLAB2*, pigmentation of labrum 2; *FWL*, forewing length; *FWW*, forewing width; *CUB A*, cubital vein, distance a; *CUB B*, cubital vein, distance b; *CI*, cubital index; *A4*, wing angle A4; *B4*: wing angle B4; *D7*, wing angle D7; *E9*, wing angle E9; *G18*, wing angle G18; *J10*, wing angle J10; *J16*, wing angle J16; *K19*, wing angle K19; *L13*, wing angle L13; *N23*, wing angle N23; *O26*, wing angle O26.

The results showed that *PT2*, *PT3*, *PT4*, *PSC1*, *PSC2*, and *B4* were moderately negatively correlated with altitude (Figure 7). *TIB*, *TAL*, *LEG*, *FWL*, *FWW*, *A4*, and wing angle N23 (*N23*) were moderately positively correlated with altitude. *FEM*, *LS3*, *WML*, *WMT*, sternite 6 longitudinal (*S6L*), sternite 6 transversal (*S6T*), *B4*, *E9*, and wing angle J10 (*J10*) were moderately positively correlated with latitude. *FEM*, *LS3*, *WML*, *S6L*, and *S6T* were moderately positively correlated with longitude.

### 3.6. Cluster Analysis of Morphologic Features

**Figure 8:**
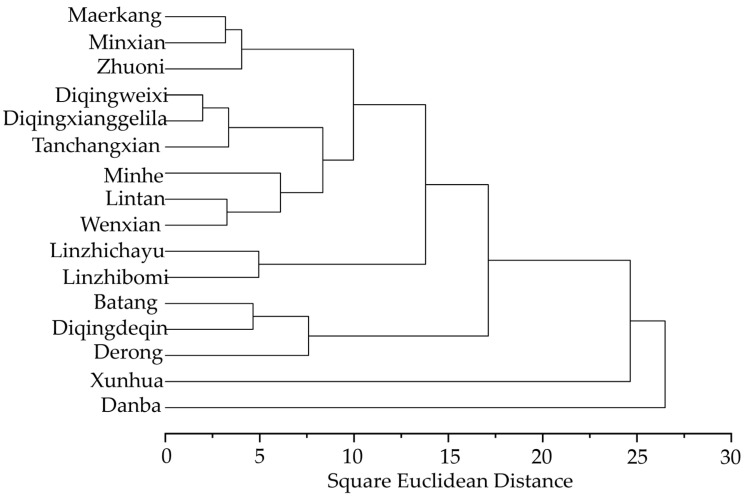
Cluster analysis plot of *A. cerana* populations based on each colony means of 30 features, including size and wing discriminant functions scores.

We found that applying different morphologic features yields different outcomes, thereby leading to potentially disparate conclusions (Figure 8, Figures S3 and S4). According to the cluster analysis result based on the means discriminant functions scores of each individual from 16 sample counties from the QTP, honey bees were differentiated into five populations (Figure 8). There were three large groups: group I (including Maerkang, Minxian, Zhuoni, Diqingweixi, Diqingxianggelila, Tanchangxian, Minhe, Lintan, Wenxian), group II (including Linzhichayu and Linzhibomi), and group III (including Batang, Derong and Diqingdeqin). The two small populations, IV and V, namely Xunhua and Danba, clustered separately. For *A. cerana* plateau colonies, the clustering results were largely related to the geographical location.

### 3.7. Stepwise Discriminant Analysis of Morphometric Features

**Figure 9:**
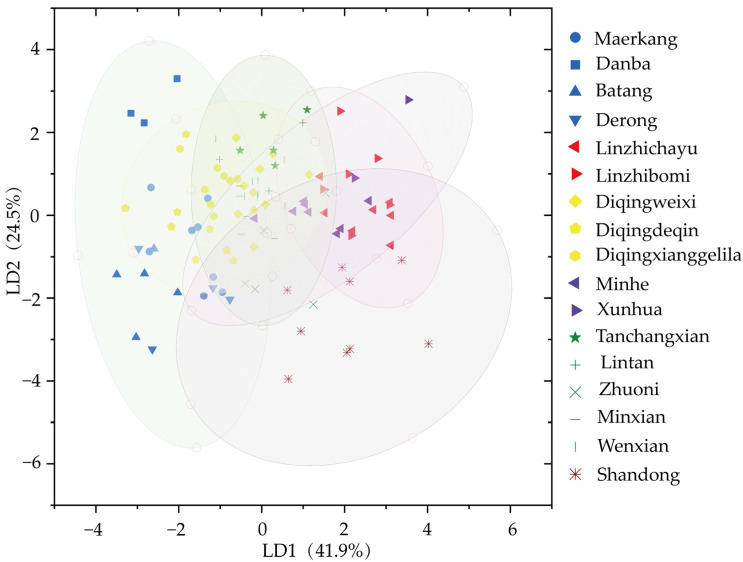
Stepwise discriminant analysis plot of *A. cerana* populations based on colony means of 40 morphometric features, with confidence ellipses at 95% (LD1 vs. LD2). Each symbol represents one population, and different provinces have different colors.

Further, we performed the stepwise discriminant analysis of morphometric features with an overall accuracy rate of 62.9% (Table S4). As shown, the first two discriminant functions (LD1 and LD2) accounted for 41.9% and 24.5% of the total variance, respectively. The samples from Shandong, forming a single cluster in the bottom right corner of the graph, are separated from other provinces (Figure 9). Predicted group membership information based on classification results showed that the correctly classified population is 44.8% after cross-verification (Table S5). Only Danba and Linzhichayu were judged exactly into corresponding populations. The samples from Yunnan, 17.24%, and Gansu, 3.70%, were wrongly judged into Sichuan. The samples from Sichuan, 14.29%, and Gansu, 22.22%, were wrongly judged into Yunnan. The samples from Yunnan, 3.45%, and Gansu, 14.81%, were wrongly judged into Qinghai. The samples from Sichuan, 4.76%, Xizang, 15.38%, Yunnan, 27.59%, and Qinghai, 20.00%, were wrongly judged into Gansu.

## 4. Discussion

*Apis cerana* is a native type of honey bee in China. Before *A. mellifera* was introduced, *A. cerana* was the only managed species that could be used to obtain bee products [3]. For many years, many bee taxonomists have conducted genetic diversity studies on *A. cerana* in different regions based on morphological differences and ecological and geographical environments [1,8,32,38,39,40,41]. Previous research showed that the western population (parts of the QTP) formed an ancestral cluster, while the MY population from Shandong was highly admixed in all cases, with large differences among individuals [38]. To explore the influence of geographical coordinate factors on the phenotypic variations in *A. cerana* in the QTP, we chose collection sites at lower altitudes from Shandong. However, due to the unique geographical conditions of the QTP, there have been no studies reporting the genetic diversity of all *A. cerana* in the region [13,24,30]. The QTP is located in southwest China, with complex terrain and a unique climate [42]. Its average elevation is 4000 m, forming many unique species resources [43]. Our study investigated the morphological diversity of *A. cerana* in the QTP region by identifying different populations based on classic morphometry, thus exploring the differentiation based on adaptive morphological characteristics and offering valuable insights for conserving and utilizing bee resources.

In this study, we analyzed the morphological features of *A. cerana* in the QTP region. Correlation analysis among 40 morphological features showed that hind leg size was highly correlated with the features of tergite and sternite. Specifically, *T3* and *T4* were highly correlated with *WML* and *WMT*. The ability of bees to collect and carry pollen was related to the characteristics of the leg. The hind leg is highly differentiated into a convenient structure for pollen collection [44]. The structure of the tibia is wide and flat, with long hair on the edge, forming a “pollen basket” specially loaded with pollen [45]. *T3* and *T4* were proportional to the worker bees’ abdomens where the honey sac is located [46]. It is reported that the storage capacity of the honey sac is proportional to the length of tergite 3 and 4, and the larger the honey sac, the stronger the honey production performance [46]. The results indicated that bees with larger abdomens and longer hind legs have strong adaptability to the relatively harsh natural environment.

The principal components analysis showed that the information on the morphology features of worker bees was mainly reflected in 12 principal components, and their cumulative contribution rate was 79.085%. According to these analyses, the morphological variation in the 16 bee populations was extremely complex. Additionally, the stepwise discriminant results proved the reliability of the above cluster analysis results and indicated that there is a large variation within the selected sampling areas of Sichuan, Yunnan, and Gansu populations. Multiple morphological features contributed to the variation among the populations, which fully indicated the rich morphological diversity of *A. cerana* in the QTP.

In the gradient change in environmental factors, the altitude gradient integrated many environmental factors, including temperature, rainfall, humidity, atmospheric composition, and light [47]. It can directly affect the body size and color of bees and the natural distribution of bee populations in different altitudes and then cause the genetic diversity of *A. cerana* [48,49]. By analyzing the correlation between environmental factors and morphological features, it was found that many morphological characteristics of the populations showed adaptation to altitude and oxygen levels. The *A. cerana* in the QTP adapted to the environment changes through morphological indicators related to the flyability (*FWL* and *FWW*), body size (*FEM*, *TIB*, *TAL*, *TAW*, *LS3*, *S6L*, and *S6T*), and wax excretion performance (*WML* and *WMT*). Similarly, Yunnan–Kweichow Plateau-type Chinese honey bees have a larger size, darker body color, and longer villi than those of eastern Central China-type populations. A large body of work shows that oxygen level influences insect body size [50,51]. Oxygen limitation defines critical weight. When insects are grown in an atmosphere with reduced oxygen, the critical weight is lower, resulting in a smaller body size [52]. As mentioned above, the one reason why the body size of honey bees in Linzhichayu and Linzhibomi is smaller than others may be the hypoxic environment of the QTP as the thin air will affect an individual’s oxygen supply [53]. Previous research showed that hypoxia always results in an extended growth phase and a smaller body Size. The under-positive selection gene coding for 6-phosphofructokinase (Pfk1, APICC_02409) is involved in the hypoxia-inducible transcription factors (HIFs) signaling pathway and has been shown to sense and respond to hypoxia [1,54,55]. In addition, air density decreases with height, providing less support for flapping changing the ideal ratio between wing size and weight for these bees [56]. Moreover, variations in latitude and longitude in many biological groups can affect the morphological characteristics of organisms [57,58]. For example, compared with the honey bees at low altitudes in Shandong, the honey bees in the QTP are larger, darker, and have stronger flying and gathering abilities. The body size of *A. cerana* increased with the northward shift in latitude, and the body color deepened with the eastward shift in longitude, which may be related to the decrease in annual temperature related to the covariation of latitude and longitude [59]. The morphological changes have important biological and ecological significance for honey bees to adapt to the environment where they live [32,40]. In order to keep warm and sustain survival, honey bees need to increase food intake and collect more pollen [9]. The indicators that reflect the body size of bees, such as legs, wings, tergites, and sternites, are also increasing. In addition, the pigmentation of the tergite, labrum, and scutellum of bees become darker, which is beneficial to absorb heat and resist cold. In order to adapt to the harsh environment, such as low temperatures and hypoxia, honey bees at high altitudes need larger wings to meet their collection needs and a stronger wax secretion basis to maintain the internal temperature of the colony. Our study demonstrated that honey bee morphology, including body size, color, and wing characteristics, is related to environmental features. With the increase in latitude, longitude, and altitude, *A. cerana* tends to have larger forewings, longer legs, and darker color, which is also the result of the environmental adaptation of *A. cerana*.

The cluster analysis based on morphology features showed that the clustering results were largely related to the geographical location of sampling sites. However, the application of color features yields different outcomes, thereby leading to potentially disparate conclusions. We consider that there is some error in the identification of *A. cerana* using the standard methods for characterizing *A. mellifera*. The morphological identification standard methods of *A. cerana* are necessary to be further improved. In addition, because honey bees were collected from unmanaged traditional or top-bar hives populated by wild swarms, without strict control of the bee age, it was impossible to distinguish between nurses and foragers. The hair of foragers fell off during foraging; therefore, it cannot reflect the normal hair length of the colonies, resulting in a not distinct rule. Something special is that the samples from Tanchangxian formed one cluster with Diqingweixi and Diqingxianggelila even though there are distant geographical locations between them. Xunhua and Danba are isolated from other populations because they are surrounded by the Yellow River or mountains, suggesting that a strong physical barrier is one of the main factors affecting population differentiation rather than simple geographical distance. Therefore, the combined effects of multiple environmental factors can affect the genetic diversity of *A. cerana* populations.

In our results, we found that compared with the honey bees in non-plateau areas, *A. cerana* in the QTP had larger wings, longer legs, deeper pigmentation, stronger flight ability, and stronger honey-collecting ability [3], which could help them adapt well to the cold and low-oxygen environment of the plateau. With the rise in the beekeeping industry in China, unrestricted introductions and hybridization have increased the conservation difficulty of native bees. *Apis cerana* in the QTP plays an indispensable role in maintaining the local ecological balance. Promoting the conservation and utilization of native *A. cerana* is the best way to protect the unique ecological environment of the QTP. Furthermore, the study also provides reference data for the study on the adaptation of *A. cerana* in high-altitude regions.

## 5. Conclusions

*Apis cerana* in the QTP has diverse morphological characteristics and rich genetic diversity. Compared with the standard method for the morphology of *A. mellifera*, there were significant differences from *A. cerana*. Only four types of labrum pigmentation were first found in this study. Moreover, environmental factors such as altitude, latitude, and longitude have important effects on the morphological diversity of *A. cerana*. In comparing the samples in Shandong, it was found that the honey bees in the QTP have larger forewings, longer legs, and darker colors with an increase in latitude, longitude, and altitude. The results of the genetic diversity of honey bees in this study can be used to understand the characteristics of bee resources in the QTP. A primary conservation strategy for *A. cerana* in the QTP should aim to maintain their integrity, including immediate intervention and prevention of rapid trait regression caused by anthropogenic activities, such as the persistent importation of even more colonies of *A. mellifera*. We advocate establishing nature reserves for *A. cerana* in the QTP and expanding the scale of *A. cerana* breeding. On this basis, the regional local good species were selected, and the government or social groups were mobilized to protect the surrounding nectar and pollen source plants to provide a good habitat environment for them.

## Data Availability

The original contributions presented in this study are included in the article/Supplementary Material. Further inquiries can be directed to the corresponding author.

## Supplementary Materials

The following supporting information can be downloaded at https://www.mdpi.com/article/10.3390/life15020255/s1, Figure S1: The comparison of the pigmentation of labrum between *A. mellifera* and *A. cerana*; Figure S2: The pigmentation of tergite 2 of *A. mellifera*; Figure S3: Cluster analysis of *A. cerana* populations based on each colony means of 37 features (including size, wing, and color) discriminant functions scores; Figure S4: Cluster analysis of *A. cerana* populations based on each colony means of all the 40 features (including hair, size, wing, and color) discriminant functions scores; Table S1: The characteristics and abbreviations of the 40 morphology features; Table S2: The mean and standard deviation of the 40 morphology features for every colony (Shandong included); Table S3: The coefficient of variation in the 40 morphology features for every colony (Shandong included); Table S4: Predicted group membership information of stepwise discriminant analysis; Table S5: Cross-verified predicted group membership information of stepwise discriminant analysis.
