# Supplementary material for: An Evaluation of Morphometric Characteristics of Honey Bee (Apis cerana) Populations in the Qinghai–Tibet Plateau in China
Source: Life (Basel). 2025 Feb 7;15(2):255. doi: 10.3390/life15020255 (PMC11856382; doi:10.3390/life15020255)
Supplement: Supplementary file 1 [file life-15-00255-s001.zip › life-3436495-supplementary.pdf]

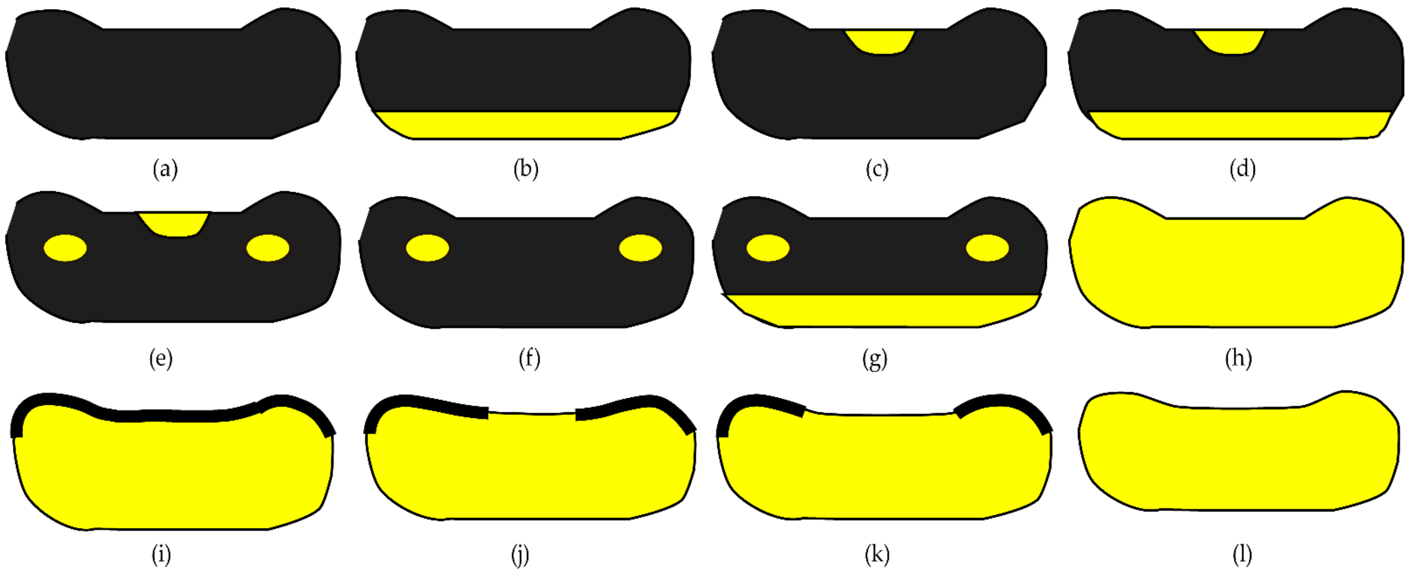

Figure S1. The comparison of the pigmentation of labrum between *A. mellifera* and *A. cerana*. The pigmentation of labrum of *A. mellifera*. (**a**) 00, labrum completely black; (**b**) 01, the yellow stripe on the bottom of the labrum; (**c**) 03, the semicircular patch on the top of the labrum; (**d**) 13, the yellow stripe on the bottom of the labrum and the semicircular patch on the top of the labrum; (**e**) 35, the semicircular patch on the top of the labrum and the two circle patches both ends of the labrum; (**f**) 35, the two circle patches both ends of the labrum; (**g**) 50, the two circle patches both ends of the labrum and the yellow stripe on the bottom of the labrum; (**h**) 60, labrum completely yellow. The pigmentation of labrum of *A. cerana*. (**i**) 53, the black extension at either end of the labrum that forms a long line; (**g**) 55, the black gradually lengthens to form a line, but the two ends of the line are not connected; (**k**) 55, little black patches on both ends of the labrum; (**l**) 60, labrum completely yellow.

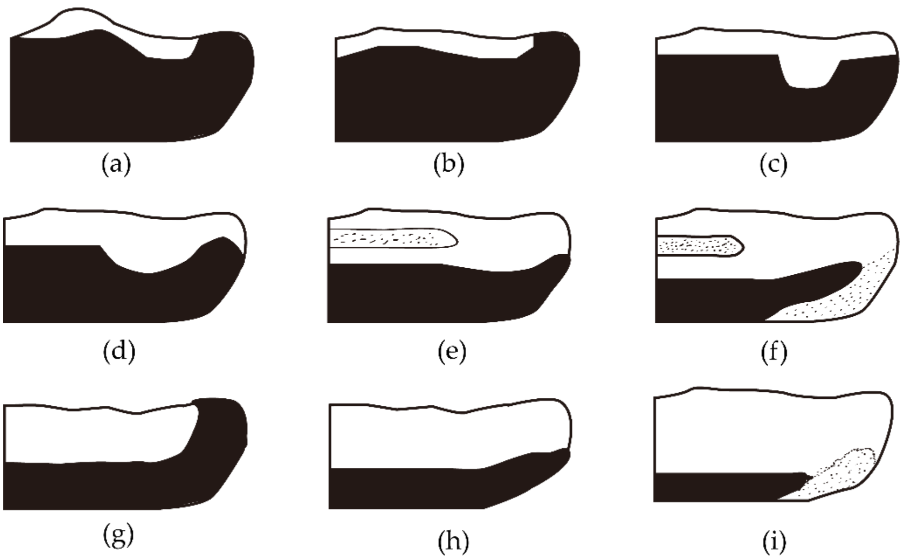

Figure S2. The pigmentation of tergite 2 of *A. mellifera*. (**a**) 1, tergite 2 almost completely dark; (**b**) 1, the light pigmentation on the upper edge of tergite 2, but not extend to the ends of tergite 2; (**c**) 3, the light pigmentation on the upper edge of tergite 2 and a protrusion of light pigmentation at the end of tergite 2; (**d**) 4, the light pigmentation at the upper edge of tergite 2 is wider than 3 and a protrusion of light pigmentation at the end of tergite 2 is bigger than 3; (**e**) 5, the medium dark pigmentation at the bottom of tergite 2 and the light pigmentation at the middle-upper of tergite 2; (**f**) 6, the medium dark pigmentation at the bottom of tergite 2 but not extend to the ends of tergite 2, and the light pigmentation at the middle-upper of tergite 2 is shorter than 5; (**g**) 7, the dark pigmentation extension at the edge of tergite 2; (**h**) 8, the dark pigmentation extension only at the bottom-edge of tergite 2 and shorter than 7; (**i**) 9, the dark pigmentation only at the bottom of tergite 2, but not extend to the ends of tergite 2.

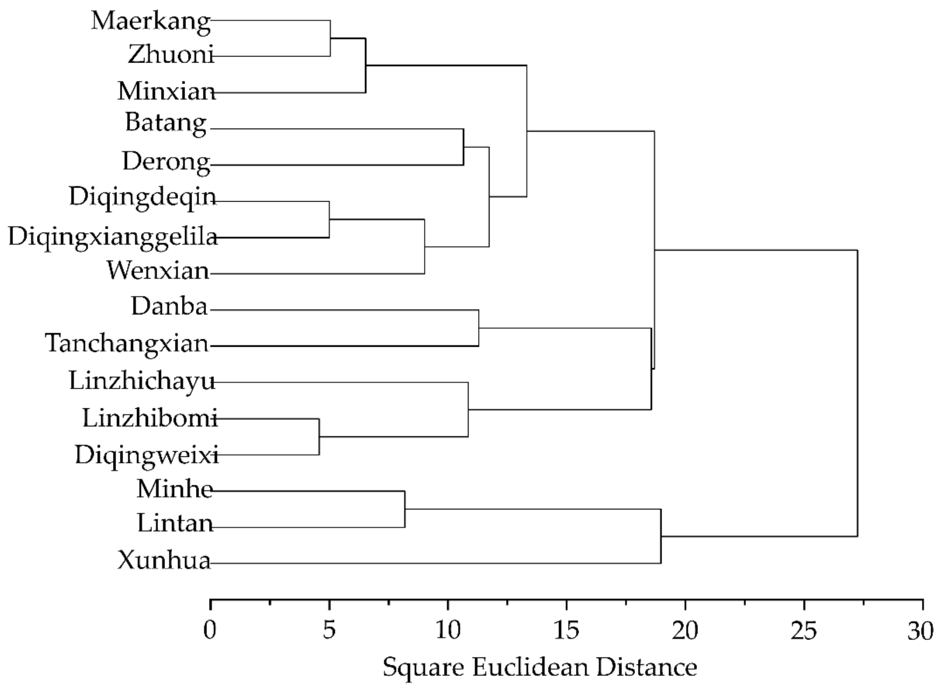

Figure S3. Cluster analysis plot of *A. cerana* populations based on each colony means of 37 features (including size, wing and color) discriminant functions scores.

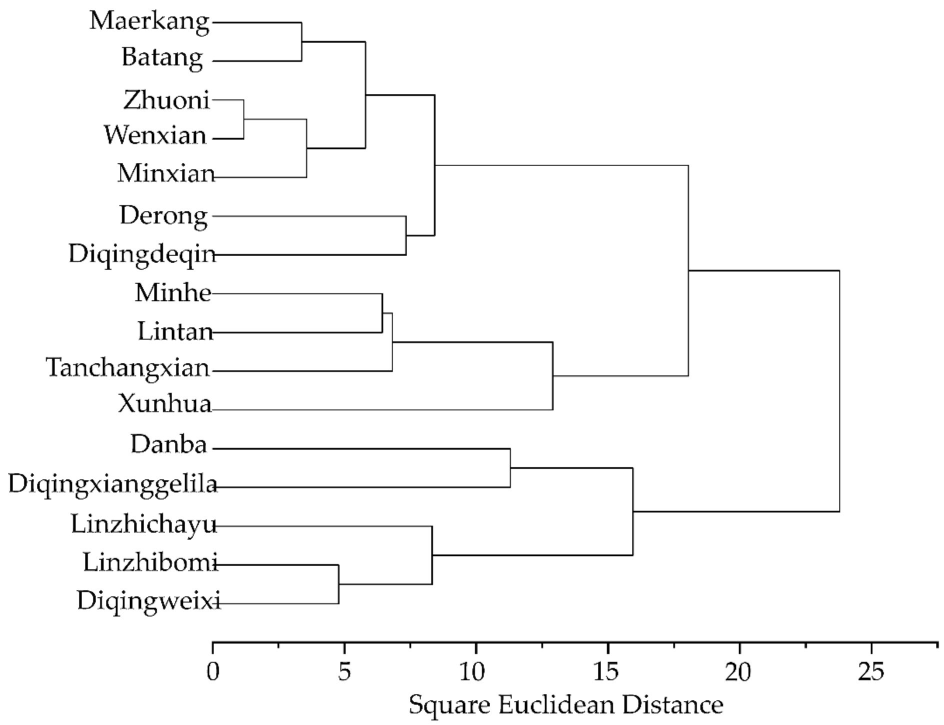

Figure S4. Cluster analysis plot of *A. cerana* populations based on each colony means of all the 40 features (including hair, size, wing and color) discriminant functions scores.

Table S1. The characters and abbreviations of the 40 morphology features.

	Characters	Abbreviations
Hair	Length of cover hair on tergite 5	*HLT5*
	Width of tomentum on tergite 4	*TOM A*
	Width of stripe posterior of tomentum	*TOM B*
Size	Femur	*FEM*
	Tibia	*TIB*
	Basitarsus length	*TAL*
	Basitarsus width	*TAW*
	Tergite 3, longitudinal	*T3*
	Tergite 4, longitudinal	*T4*
	Sternite 3, longitudinal	*LS3*
	Wax mirror of sternite 3 longitudinal	*WML*
	Wax mirror of sternite 3, transversal	*WMT*
	Distance between wax mirrors st. 3	*WD*
	Sternite 6, longitudinal	*S6L*
	Sternite 6, transversal	*S6T*
	Length of hind leg	*LEG*
	Tergite 3 and 4, longitudinal	*T3+4*
Color	Pigmentation of tergite 2	*PT2*
	Pigmentation of tergite 3	*PT3*
	Pigmentation of tergite 4	*PT4*
	Pigmentation of scutellum, Cupola	*PSC1*
	Pigmentation of scutellum, B and K	*PSC2*
	Pigmentation of labrum 1	*PLAB1*
	Pigmentation of labrum 2	*PLAB2*
Wing	Forewing length	*FWL*
	Forewing width	*FWW*
	Cubital vein, distance a	*CUB A*
	Cubital vein, distance b	*CUB B*
	Cubital index	*CI*
	Wing angle A4	*A4*
	Wing angle B4	*B4*
	Wing angle D7	*D7*
	Wing angle E9	*E9*
	Wing angle G18	*G18*
	Wing angle J10	*J10*
	Wing angle J16	*J16*
	Wing angle K19	*K19*
	Wing angle L13	*L13*
	Wing angle N23	*N23*
	Wing angle O26	*O26*

Table S2. The mean and standard deviation of the 40 morphology features for every colony (Shandong included).

Features	Maerkang	Danba	Batang	Derong	Linzhichayu	Linzhibomi
HLT5	82.09±16.67^{ab}	88.5±16.04^{ab}	87.05±13.27^{ab}	88.68±4.68^{ab}	65.16±9.02^{b}	80.99±15.69^{ab}
TOM A	48.8±27.14^{abc}	40.03±6.95^{abc}	39.2±19.07^{abc}	24.97±1.76^{abc}	58.5±15.32^{a}	42.03±19.14^{abc}
TOM B	41.2±24.81^{abc}	60.55±0.72^{a}	45.36±17.3^{abc}	50.36±15.12^{abc}	22.2±20.37^{bc}	39.98±21.53^{abc}
FEM	249.55±5.32^{abcde}	244.05±0.69^{cde}	253.39±5.98^{abcd}	250.54±2.57^{abcd}	239.14±3.9^{cd}	243.37±2.46^{de}
TIB	313.22±7.77^{abc}	310.13±2.28^{abcd}	321.2±2.39^{a}	318.13±5^{ab}	300.31±3.55^{a}	304.7±3.77^{d}
TAL	199.95±3.73^{abc}	195.09±2.51^{bcd}	200.64±3.57^{abc}	201.65±2.02^{ab}	189.51±3.09^{b}	192.16±2.98^{cd}
TAW	108.86±2.36^{ab}	106.38±2.97^{b}	108.8±2.41^{ab}	109.77±1.28^{ab}	105.13±1.97^{b}	106.63±1.65^{b}
T3	194.89±9.37^{a}	191±5.29^{ab}	192.33±4.8^{ab}	196±2.16^{a}	189.4±2.3^{ab}	190.25±4.33^{ab}
T4	187.25±9.42^{a}	184.3±0.48^{a}	185.97±4.11^{a}	190.86±2.27^{a}	181.7±2.24^{a}	186.63±10.31^{a}
LS3	259.52±5.32^{a}	245.28±3.09^{de}	259.33±6.9^{a}	256.55±4.97^{abcd}	242.13±3.42^{e}	246.32±3.24^{cde}
WML	124.48±4.24^{abc}	115.29±4.04^{e}	126.46±4.47^{a}	123.1±2.76^{abcd}	117.89±1.42^{cde}	116.67±5.39^{de}
WMT	230.61±5.95^{abcd}	220.72±5.85^{de}	232.66±6.76^{ab}	232.32±4.07^{abc}	217.92±3.21^{e}	221.68±2.58^{bce}
WD	24.86±2.42^{ab}	28.87±1.81^{ab}	22.84±1.75^{bc}	24.91±1.74a^{b}	26.87±4.61^{ab}	27.2±2.54^{ab}
S6L	238.34±4.71^{a}	232.43±2.98^{a}	240.25±2.98^{a}	235.64±4.93^{a}	221.6±2.02^{a}	226.2±3.62^{a}
S6T	288.44±5.37^{a}	284.05±9.61^{a}	293.45±7.33^{a}	288.36±3.75^{a}	269.38±4.35^{a}	273.59±5.12^{a}
LEG	762.71±15.23^{abc}	749.28±5.39^{abcd}	775.23±9.53^{ab}	770.32±8.34^{ab}	728.97±7.8^{d}	740.23±7.82^{cd}
T3+4	382.14±18.45^{ab}	375.3±5.74^{ab}	378.3±8.85^{ab}	386.86±4.43^{a}	371.1±1.63^{ab}	376.88±13.99^{ab}
PT2	4.81±1.36^{bcdef}	4.23±2.49^{cdefg}	3.2±1.5^{defgh}	1.78±0.9^{fgh}	7.68±0.79^{ab}	7.19±1.12^{abc}
PT3	6.58±0.68^{abcd}	6.93±0.12^{abc}	6.28±0.74^{bcd}	5.68±0.81^{cd}	7.7±0.2^{ab}	7.13±0.52^{abc}
PT4	5.88±0.91^{bc}	5.63±1.1^{bc}	5.57±0.56^{bc}	5.78±0.94^{bc}	7.02±0.6^{ab}	5.69±0.8^{bc}
PSC1	6.37±0.29^{ab}	6.17±1.18^{ab}	3.07±2.61^{bc}	3.83±1.85^{abc}	7.12±0.67^{a}	6.16±0.54^{ab}
PSC2	1.53±0.42^{abcd}	1.8±0.35^{abc}	0.5±0.8^{cd}	0.9±0.62^{abcd}	2.52±1.15^{a}	1.48±0.7^{abcd}
PLAB1	5.21±0.15^{b}	5.23±0.21^{b}	5.17±0.15^{b}	5.25±0.06^{b}	5.18±0.22^{b}	5.13±0.12^{b}
PLAB2	3.9±0.59^{ab}	3.8±0.26^{ab}	4.08±0.67^{ab}	3.4±0.32^{b}	3.78±1.06^{ab}	3.91±0.54^{ab}
FWL	880.75±15.22^{ab}	871.74±8.49^{abcd}	889.33±14.94^{a}	880.85±3.87^{ab}	831.17±7.01^{e}	843.31±10.44^{cde}
FWW	311.77±3.54^{abc}	307.93±2.48^{abc}	311.15±7.39^{abc}	311.95±1.6^{abc}	294.63±2.63^{e}	296±4.22^{de}
CUB A	63.01±17.81^{a}	57.73±1.94^{a}	54.33±1.8^{a}	57.76±2.28^{a}	53.02±1.77^{a}	54.38±2.37^{a}
CUB B	15.22±5.37^{ab}	14.53±0.63^{ab}	18.17±1.09^{a}	16.56±1.55^{ab}	13.66±0.45^{ab}	13.41±0.99^{ab}
CI	4.35±0.6^{abc}	4.1±0.3^{abc}	3.09±0.27^{d}	3.59±0.4^{bcd}	3.98±0.11^{abcd}	4.22±0.44^{abc}
A4	33.68±0.86^{ab}	32.34±0.89^{abcd}	33.49±0.95^{abc}	32.47±0.87^{abcd}	32.17±0.93^{abcd}	32.31±1.06^{abcd}
B4	106.82±1.76^{cd}	106.97±1.13^{cd}	105.78±1.55^{cd}	105.33±1.87^{cd}	108.96±2.09^{bc}	107.84±1.91^{cd}
D7	91.93±0.84^{cd}	91.26±1.16^{d}	94.94±1.45^{abcd}	92.62±1.2^{cd}	95.2±1.89^{abc}	95.33±1.94^{abc}
E9	19.82±0.29^{ab}	19.55±0.91^{ab}	19.69±0.41^{ab}	20.08±0.54^{a}	18.89±0.49^{ab}	18.71±0.87^{ab}
G18	88.05±1.84^{d}	88.72±0.25^{bcd}	90.39±1.45^{abcd}	88.8±1.78^{bcd}	88.38±1.37^{cd}	88.53±1.49^{bcd}
J10	45.48±0.67^{ab}	47.12±2.23^{a}	44.77±1.16^{ab}	43.55±1.86^{b}	44.9±2.17^{ab}	46.1±1.88^{ab}
J16	103.3±2.37^{abc}	103.02±1.08^{abc}	101.99±2.07^{abc}	99.39±1.94^{c}	103.29±1.08^{abc}	100.06±1.96^{bc}
K19	80.59±1.28^{bc}	84.3±0.94^{a}	80.83±1.44^{bc}	77.9±1.45^{c}	77.86±0.67^{c}	80.58±2.05^{bc}
L13	15.54±0.64^{a}	14±0.94^{ab}	14.59±0.59^{ab}	14.64±1.07^{ab}	15.08±0.67^{ab}	14.47±0.51^{ab}
N23	84.82±2.16^{a}	84.51±1.4^{a}	84.01±1.31^{a}	83.48±3.15^{a}	84.34±1.95^{a}	82.35±1.63^{a}
O26	28.92±0.84^{b}	33.69±0.49^{a}	33.07±1.58^{a}	31.79±3.72^{ab}	32.06±2.33^{ab}	33.02±2.04^{ab}

Note: The measured value (*HLT5, TOM A, TOM B, FEM, TIB, TAL, TAW, T3, T4, LS3, WML, WMT, WD, S6L, S6T, LEG, T3+4, FWL, FWW, CUB A, CUB B, CI*) is 100 times the actual value (mm). Others are the actual value. Values with the same letter are not significantly different from each other, otherwise they are significant ($p< 0.05$).

Features	Diqingweixi	Diqingdeqin	Diqingxianggelila	Minhe	Xunhua	Tanchangxian
HLT5	82.2±15.69^{ab}	69.03±6.26^{b}	66.71±8.7^{b}	79.64±17.79^{ab}	63.29±1.99^{b}	86.17±12.92^{ab}
TOM A	32.48±7.26^{abc}	24.35±9.87^{abc}	54.4±24.91^{ab}	27.26±4.28^{abc}	32.52±1.88^{abc}	20.86±12.43^{bc}
TOM B	58.42±7.61^{ab}	61.69±2.62^{a}	41.28±27^{abc}	60.87±4.99^{a}	54.47±0.83^{abc}	54.79±28.13^{abc}
FEM	244.44±5.3^{cde}	254.49±3.34^{abc}	246.57±2.83^{bcde}	257.44±4.47^{a}	249.85±4.46^{abcd}	252.8±3.8^{abcd}
TIB	310.07±4.47^{abcd}	318.84±5.51^{ab}	307.99±5.83^{bcd}	318.79±6.73^{ab}	310.15±7.76^{abcd}	315.73±6.41^{abc}
TAL	196.27±3.17^{abcd}	205.01±2.48^{a}	198.27±4.54^{abcd}	197.67±4.78^{abcd}	195.24±1.45^{bcd}	197.26±5.4^{abcd}
TAW	107.99±2.85^{ab}	112.48±2.36^{a}	107.99±2.71^{ab}	108.13±1.06^{ab}	107.93±2.61^{ab}	107.91±2.61^{ab}
T3	189.19±6.66^{ab}	194.33±4.5^{a}	189.13±4.12^{ab}	190.5±7.42^{ab}	179±1.41^{b}	188±5.83^{ab}
T4	182.86±6.43^{a}	189.44±4.55^{a}	183.52±4.88^{a}	188.31±9.29^{a}	175.57±2.96^{a}	182.97±7.93^{a}
LS3	247.83±5.54^{bcde}	254.17±2.72^{abcd}	249.65±2.66^{abcde}	256.92±4.18^{abc}	250.7±3.72^{abcde}	249.46±5.38^{abcde}
WML	118.44±2.79^{bcde}	123.23±2.02^{abcd}	118.51±3.13^{bcde}	124.24±3.76^{abcd}	120.17±0.77^{abcde}	121.4±2.93^{abcde}
WMT	223.37±4.49^{abcde}	227.55±2.04^{abcde}	223.03±4.44^{bcde}	228.3±7.09^{abcde}	217.75±2.58^{e}	228.3±5.06^{abcde}
WD	26.99±3.16^{ab}	27.2±2.76^{ab}	29.72±3.61^{a}	25.52±3.67^{ab}	17.08±0.5^{c}	26.29±1.46^{ab}
S6L	228.99±4.17^{a}	232.6±3.65^{a}	230.55±4.83^{a}	241.12±6.64^{a}	229.38±4.35^{a}	231.89±4.02^{a}
S6T	277.94±5.37^{a}	283.51±2.96^{a}	279.88±5.63^{a}	287.69±9.49^{a}	276.62±1.82^{a}	280.75±6.73^{a}
LEG	750.78±10.58^{abcd}	778.34±7.88^{a}	752.83±11.91^{abcd}	773.9±15.76^{ab}	755.24±13.68^{abcd}	765.79±14.68^{abc}
T3+4	372.05±12.95^{ab}	383.78±8.26^{ab}	372.65±8.92^{ab}	378.81±15.79^{ab}	354.57±4.37^{ab}	333.37±83.79^{b}
PT2	6.16±1.14^{abcd}	4.13±1.47^{defgh}	5.16±1.09^{bcde}	1.33±1.58^{gh}	4.8±1.13^{bcdef}	3.28±2.01^{defgh}
PT3	6.79±0.43^{abcd}	6.03±1.03^{cd}	7.1±0.56^{abc}	6.18±0.93^{bcd}	5.85±0.21^{cd}	6.6±0.89^{abcd}
PT4	5.36±0.67^{bc}	5.72±0.96^{bc}	6.34±1.03^{abc}	5.6±0.43^{bc}	5±0^{c}	5.78±0.86^{bc}
PSC1	4.81±1.45^{abc}	1.87±1.93^{c}	4.46±2.28^{abc}	4.55±2.45^{abc}	4.4±1.13^{abc}	4.44±2.36^{abc}
PSC2	1.42±0.79^{abcd}	0.28±0.42^{abcd}	0.93±0.89^{abcd}	0.55±0.67^{cd}	0.8±1.13^{bcd}	0.6±0.66^{cd}
PLAB1	5.07±0.11^{b}	5.17±0.1^{b}	5.25±0.2^{b}	5±0^{b}	5±0^{b}	5.06±0.09^{b}
PLAB2	4.41±0.56^{ab}	4.25±0.5^{ab}	3.91±1.03^{ab}	3.85±0.7^{ab}	4.3±0.71^{ab}	4.14±0.72^{ab}
FWL	864.8±9.27^{abcd}	878.92±8.75^{ab}	873.76±6.85^{abc}	880.68±17.28^{ab}	855.15±10.41^{bcde}	873.47±22.45^{abc}
FWW	305.28±4.5^{bcde}	309.26±5.49^{abc}	308.78±3.6^{abc}	315.24±6.16^{ab}	307.59±10.67^{abc}	311.06±8.12^{abc}
CUB A	59.21±12.96^{a}	55.23±1.17^{a}	56.99±3.27^{a}	55.31±0.78^{a}	54.31±1^{a}	55.53±1.97^{a}
CUB B	15.03±2.98^{ab}	16.26±1.66^{ab}	15.19±1.7^{ab}	12.78±0.6^{b}	12.01±1.15^{b}	14.54±1.74^{ab}
CI	4.08±0.45^{abc}	3.51±0.32^{cd}	3.91±0.54^{abcd}	4.48±0.29^{abc}	4.8±0.62^{a}	4.04±0.54^{abcd}
A4	32.5±1.12^{abcd}	33.91±0.69^{a}	32.59±1.2^{abcd}	31.41±0.57^{cd}	30.46±0.32^{d}	32.05±0.8^{abcd}
B4	106.58±2.32^{cd}	104.21±2.13^{d}	105.49±2.95^{cd}	113.27±0.97^{ab}	115.37±0.17^{a}	108.74±1.48^{bcd}
D7	94.56±1.71^{abcd}	95.14±1.55^{abc}	94.11±1.23^{bcd}	96.81±0.92^{ab}	98.37±0.79^{a}	95.68±2.36^{abc}
E9	18.94±0.66^{ab}	18.94±0.8^{ab}	18.39±0.81^{b}	19.59±0.47^{ab}	18.79±0.39^{ab}	19.08±0.42^{ab}
G18	88.53±1.68^{bcd}	89.13±1.53^{abcd}	88.23±1.34^{cd}	91.73±2.26^{abc}	92.47±0.83^{a}	88.07±1.68^{d}
J10	44.89±1.83^{ab}	45.67±1.81^{ab}	45.35±1.6^{ab}	45.41±1.04^{ab}	44.66±0.14^{ab}	46.59±2.19^{ab}
J16	101.19±2.43^{abc}	104.21±2.1^{ab}	103.67±2.04^{abc}	100.47±2.14^{bc}	105.15±0.77^{a}	103±2.58^{abc}
K19	79.68±1.47^{bc}	80.69±1.43^{bc}	80.09±1.92^{bc}	79.29±0.96^{bc}	82.48±0.78^{ab}	80.71±1.36^{bc}
L13	14.35±0.68^{ab}	14.88±0.83^{ab}	14.36±0.9^{ab}	14.15±1.04^{ab}	14.58±0.39^{ab}	13.81±0.73^{b}
N23	82.65±2.18^{a}	85.38±0.69^{a}	85.01±2.81^{a}	84.72±2.39^{a}	84.06±1.67^{a}	83.31±1.84^{a}
O26	33.96±1.9^{a}	31.93±1.19^{ab}	33.19±2.81^{a}	32.53±1.47^{ab}	32.1±1.09^{ab}	32.7±1.18^{ab}

Note: The measured value (*HLT5, TOM A, TOM B, FEM, TIB, TAL, TAW, T3, T4, LS3, WML, WMT, WD, S6L, S6T, LEG, T3+4, FWL, FWW, CUB A, CUB B, CI*) is 100 times the actual value (mm). Others are the actual value. Values with the same letter are not significantly different from each other, otherwise they are significant ($p < 0.05$).

Features	Lintan	Zhuoni	Minxian	Wenxian	Shandong
HLT5	73.78±18.3^{b}	80.4±13.43^{ab}	75.88±19.21^{ab}	105.76±9.82^{a}	79.24±8.35^{ab}
TOM A	19.68±10.75^{c}	35.09±12.61^{abc}	30.01±11.88^{abc}	52.13±11.39^{abc}	29.22±2.34^{abc}
TOM B	54.83±12.24^{abc}	54.43±5.56^{abc}	56.73±10.6^{abc}	48.92±8.49^{abc}	21.86±13.04^{c}
FEM	253.5±3.41^{abcd}	254.59±3.62^{abc}	254.18±3.11^{abc}	255.29±3.61^{ab}	247.19±8.72^{abcde}
TIB	317.71±5.67^{ab}	316.07±6.96^{abc}	317.48±1.89^{ab}	315.1±3.63^{abc}	307.91±9.01^{bcd}
TAL	198.74±2.94^{abcd}	200±4.73^{abc}	199.44±3.86^{abc}	199.86±4.83^{abc}	193.75±6.61^{bcd}
TAW	108.56±1.82^{ab}	108.18±1.96^{ab}	109.78±2.48^{ab}	109.52±4.02^{ab}	107.69±3.55^{ab}
T3	192.33±5.51^{ab}	190.33±5.24^{ab}	193.11±8.07^{ab}	195.2±8.04^{b}	197.29±6.2^{a}
T4	187.07±3.03^{a}	184.02±4.62^{a}	187.06±9.16^{a}	190.47±8.92^{a}	176.9±31.52^{a}
LS3	256.18±4.43^{abcd}	258.82±8.73^{ab}	257.3±3.12^{abc}	252.86±1.86^{abcde}	256.4±7.05^{abcd}
WML	125.76±2.96^{ab}	126.53±3.74^{a}	125.86±2.73^{ab}	125.2±2.67^{abc}	119.28±3.2^{abcde}
WMT	228.81±1^{abcd}	231.4±5.31^{abc}	233.77±5.02^{a}	232.65±3.32^{ab}	228.27±5.4^{abcde}
WD	24.34±3.26^{ab}	26.97±3.57^{ab}	25.44±1.89^{ab}	24.6±1.21^{ab}	27.41±2.77^{ab}
S6L	239.74±3^{a}	240.2±5.25^{a}	237.09±4.17^{a}	233.6±1.45^{a}	276.98±117.13^{a}
S6T	290.37±3.77^{a}	288.51±5.08^{a}	292.74±6.37^{a}	286.69±4.53^{a}	348.99±148.12^{a}
LEG	769.95±11.73^{ab}	770.65±14.82^{ab}	771.1±7.95^{ab}	770.25±9.31^{ab}	748.85±24.14^{bcd}
T3+4	379.4±8.51^{ab}	374.35±9.45^{ab}	380.18±16.99^{ab}	385.67±16.38^{ab}	374.19±33.93^{ab}
PT2	1.1±0.75^{h}	3.4±1.73^{defgh}	3.06±2.02^{efgh}	2.94±0.87^{efgh}	8.8±0.24^{h}
PT3	5.77±1.37^{ab}	5.27±1.03^{d}	6.12±0.88^{cd}	6.6±0.55^{abcd}	7.86±0.26^{a}
PT4	4.73±0.64^{c}	4.95±1.27^{c}	5.93±0.7^{bc}	6.36±0.97^{abc}	7.83±0.16^{a}
PSC1	2.8±1.51^{bc}	4.5±2.3^{abc}	3.36±1.5^{bc}	5.44±1.17^{abc}	7.14±1.01^{a}
PSC2	0±0^{d}	1.12±1.15^{abcd}	0.89±0.49^{abcd}	0.67±0.75^{cd}	2.38±0.73^{ab}
PLAB1	5±0^{b}	5.12±0.15^{b}	5.08±0.11^{b}	5.1±0.12^{b}	6±0^{a}
PLAB2	5±0^{a}	4.13±0.45^{ab}	4.1±0.6^{ab}	3.7±1.57^{ab}	0±0^{c}
FWL	880.71±4.63^{ab}	863.01±35.24^{abcd}	875.64±3.42^{ab}	866.3±6.95^{abcd}	840.35±16.5^{de}
FWW	317.46±2.05^{a}	307.15±8.54^{abcd}	308.9±2.84^{abc}	305.84±3.72^{bcde}	303.44±5.62^{cde}
CUB A	55.25±1.46^{a}	57.23±2.93^{a}	56.55±1.33^{a}	53.9±2.33^{a}	54.57±2.28^{a}
CUB B	13±1.11^{b}	13.35±0.98^{ab}	13.03±1.05^{b}	13.26±0.95^{ab}	15.59±1.56^{ab}
CI	4.47±0.3^{abc}	4.46±0.37^{abc}	4.52±0.35^{ab}	4.2±0.51^{abc}	3.63±0.5^{bcd}
A4	32.9±0.39^{abc}	32.2±0.92^{abcd}	32.49±0.91^{abcd}	33.18±1.29^{abc}	31.56±1.02^{bcd}
B4	108.47±0.97^{cd}	108.02±2.98^{cd}	107.95±2.74^{cd}	107.6±1.52^{cd}	107.42±1.44^{cd}
D7	94.84±1.07^{abcd}	93.71±2.93^{bcd}	94.39±2^{bcd}	95.38±2.24^{abc}	95.01±1.35^{abcd}
E9	19.6±0.61^{ab}	19.5±1.26^{ab}	19.44±0.83^{ab}	18.95±0.47^{ab}	19.86±0.59^{ab}
G18	90.61±1.75^{abcd}	91.98±1.02^{ab}	89.4±1^{abcd}	90.85±2.21^{abcd}	88.77±1.57^{bcd}
J10	45.56±1.24^{ab}	45.48±0.86^{ab}	45.71±1.09^{ab}	46.11±0.53^{ab}	47.89±1.38^{a}
J16	103.65±1.13^{abc}	101.44±2.41^{abc}	102.61±1.72^{abc}	103.12±1.39^{abc}	100.71±1.79^{abc}
K19	80.99±0.78^{bc}	79.69±1.13^{bc}	80±1.02^{bc}	81.76±1.1^{ab}	79.67±1.79^{bc}
L13	14.77±0.31^{ab}	14.09±0.6^{ab}	14.15±0.54^{ab}	15.42±0.98^{ab}	14.12±0.75^{ab}
N23	84.11±1.35^{a}	83.61±2.02^{a}	84.79±2.4^{a}	83.14±1.32^{a}	81.46±1.3^{a}
O26	31.27±0.71^{ab}	33.44±1.56^{a}	33.06±1.33^{a}	33.66±0.88^{a}	33.79±1.83^{a}

Note: The measured value (*HLT5, TOM A, TOM B, FEM, TIB, TAL, TAW, T3, T4, LS3, WML, WMT, WD, S6L, S6T, LEG, T3+4, FWL, FWW, CUB A, CUB B, CI*) is 100 times the actual value (mm). Others are the actual value. Values with the same letter are not significantly different from each other, otherwise they are significant ($p < 0.05$).

Table S3. The coefficient of variation of the 40 morphology features for every colony (Shandong included).

Features	Maerkang	Danba	Batang	Derong	Linzhichayu	Linzhibomi
HLT5	0.2030	0.1813	0.1524	0.0528	0.1385	0.1937
TOM A	0.5561	0.1736	0.4864	0.0705	0.2618	0.4553
TOM B	0.6022	0.0119	0.3815	0.3003	0.9176	0.5385
FEM	0.0213	0.0028	0.0236	0.0103	0.0163	0.0101
TIB	0.0248	0.0074	0.0075	0.0157	0.0118	0.0124
TAL	0.0187	0.0129	0.0178	0.0100	0.0163	0.0155
TAW	0.0217	0.0279	0.0221	0.0117	0.0188	0.0155
T3	0.0481	0.0277	0.0250	0.0110	0.0122	0.0228
T4	0.0503	0.0026	0.0221	0.0119	0.0123	0.0552
LS3	0.0205	0.0126	0.0268	0.0194	0.0141	0.0132
WML	0.0341	0.0350	0.0354	0.0224	0.0120	0.0462
WMT	0.0258	0.0265	0.0291	0.0175	0.0147	0.0116
WD	0.0973	0.0626	0.0767	0.0699	0.1715	0.0935
S6L	0.0198	0.0128	0.0124	0.0209	0.0091	0.0160
S6T	0.0186	0.0338	0.0250	0.0130	0.0161	0.0187
LEG	0.0200	0.0072	0.0123	0.0108	0.0107	0.0106
T3+4	0.0483	0.0153	0.0234	0.0114	0.0044	0.0371
PT2	0.2824	0.5883	0.4673	0.5047	0.1031	0.1557
PT3	0.1039	0.0167	0.1184	0.1420	0.0260	0.0734
PT4	0.1549	0.1947	0.1001	0.1627	0.0857	0.1402
PSC1	0.0451	0.1921	0.8497	0.4827	0.0944	0.0876
PSC2	0.2709	0.1925	1.5950	0.6909	0.4545	0.4735
PLAB1	0.0295	0.0398	0.0291	0.0110	0.0419	0.0227
PLAB2	0.1522	0.0696	0.1650	0.0930	0.2808	0.1383
FWL	0.0173	0.0097	0.0168	0.0044	0.0084	0.0124
FWW	0.0114	0.0081	0.0238	0.0051	0.0089	0.0143
CUBA	0.2827	0.0335	0.0331	0.0395	0.0333	0.0435
CUBB	0.3526	0.0435	0.0601	0.0937	0.0326	0.0741
CI	0.1389	0.0721	0.0859	0.1113	0.0272	0.1033
A4	0.0256	0.0274	0.0284	0.0268	0.0290	0.0328
B4	0.0165	0.0106	0.0146	0.0178	0.0192	0.0177
D7	0.0091	0.0127	0.0152	0.0129	0.0199	0.0204
E9	0.0144	0.0466	0.0209	0.0271	0.0260	0.0466
G18	0.0210	0.0029	0.0161	0.0200	0.0155	0.0168
J10	0.0148	0.0472	0.0258	0.0427	0.0483	0.0408
J16	0.0229	0.0105	0.0202	0.0195	0.0104	0.0196
K19	0.0159	0.0112	0.0178	0.0187	0.0086	0.0255
L13	0.0409	0.0674	0.0405	0.0731	0.0441	0.0350
N23	0.0255	0.0166	0.0156	0.0377	0.0231	0.0198
O26	0.0291	0.0147	0.0477	0.1170	0.0726	0.0618

Note: Except the samples from Shandong, the minimum values are shown in blue. The maximum values are shown in red.

Features	Diqingweixi	Diqingdeqin	Diqingxianggelila	Minhe	Xunhua	Tanchangxian
HLT5	0.1908	0.0906	0.1305	0.2234	0.0314	0.1499
TOM A	0.2236	0.4055	0.4578	0.1569	0.0578	0.5960
TOM B	0.1302	0.0425	0.6540	0.0820	0.0152	0.5135
FEM	0.0217	0.0131	0.0115	0.0173	0.0179	0.0150
TIB	0.0144	0.0173	0.0189	0.0211	0.0250	0.0203
TAL	0.0161	0.0121	0.0229	0.0242	0.0075	0.0274
TAW	0.0264	0.0210	0.0251	0.0098	0.0242	0.0242
T3	0.0352	0.0232	0.0218	0.0389	0.0079	0.0310
T4	0.0351	0.0240	0.0266	0.0494	0.0168	0.0433
LS3	0.0224	0.0107	0.0107	0.0163	0.0148	0.0216
WML	0.0236	0.0164	0.0264	0.0302	0.0064	0.0241
WMT	0.0201	0.0090	0.0199	0.0311	0.0119	0.0222
WD	0.1170	0.1013	0.1215	0.1438	0.0293	0.0556
S6L	0.0182	0.0157	0.0210	0.0276	0.0190	0.0173
S6T	0.0193	0.0104	0.0201	0.0330	0.0066	0.0240
LEG	0.0141	0.0101	0.0158	0.0204	0.0181	0.0192
T3+4	0.0348	0.0215	0.0240	0.0417	0.0123	0.2513
PT2	0.1843	0.3561	0.2107	1.1939	0.2357	0.6129
PT3	0.0632	0.1709	0.0782	0.1504	0.0363	0.1355
PT4	0.1240	0.1679	0.1618	0.0771	0.0000	0.1490
PSC1	0.3015	1.0336	0.5103	0.5376	0.2571	0.5305
PSC2	0.5593	1.4877	0.9613	1.2106	1.4142	1.1055
PLAB1	0.0213	0.0200	0.0381	0.0000	0.0000	0.0177
PLAB2	0.1269	0.1169	0.2640	0.1806	0.1644	0.1738
FWL	0.0107	0.0100	0.0078	0.0196	0.0122	0.0257
FWW	0.0147	0.0178	0.0117	0.0195	0.0347	0.0261
CUB A	0.2188	0.0213	0.0573	0.0142	0.0185	0.0356
CUB B	0.1985	0.1021	0.1118	0.0473	0.0961	0.1199
CI	0.1106	0.0911	0.1378	0.0652	0.1282	0.1337
A4	0.0343	0.0203	0.0367	0.0181	0.0106	0.0250
B4	0.0218	0.0205	0.0279	0.0086	0.0014	0.0136
D7	0.0181	0.0163	0.0130	0.0095	0.0081	0.0247
E9	0.0347	0.0425	0.0442	0.0237	0.0205	0.0221
G18	0.0190	0.0172	0.0152	0.0247	0.0090	0.0191
J10	0.0408	0.0396	0.0352	0.0229	0.0030	0.0470
J16	0.0241	0.0201	0.0196	0.0213	0.0073	0.0250
K19	0.0185	0.0177	0.0240	0.0121	0.0095	0.0169
L13	0.0474	0.0557	0.0628	0.0733	0.0270	0.0529
N23	0.0264	0.0081	0.0331	0.0282	0.0199	0.0221
O26	0.0561	0.0374	0.0847	0.0453	0.0341	0.0362

Note: Except the samples from Shandong, the minimum values are shown in blue. The maximum values are shown in red.

Features	Lintan	Zhuoni	Minxian	Wenxian	Shandong
HLT5	0.2480	0.1670	0.2532	0.0928	0.1053
TOM A	0.5463	0.3594	0.3959	0.2185	0.0800
TOM B	0.2232	0.1021	0.1869	0.1736	0.5968
FEM	0.0135	0.0142	0.0122	0.0141	0.0353
TIB	0.0179	0.0220	0.0060	0.0115	0.0293
TAL	0.0148	0.0236	0.0194	0.0241	0.0341
TAW	0.0168	0.0181	0.0225	0.0367	0.0330
T3	0.0286	0.0275	0.0418	0.0412	0.0314
T4	0.0162	0.0251	0.0489	0.0468	0.1782
LS3	0.0173	0.0337	0.0121	0.0074	0.0275
WML	0.0235	0.0295	0.0217	0.0213	0.0268
WMT	0.0044	0.0230	0.0215	0.0143	0.0237
WD	0.1340	0.1325	0.0742	0.0491	0.1012
S6L	0.0125	0.0218	0.0176	0.0062	0.4229
S6T	0.0130	0.0176	0.0218	0.0158	0.4244
LEG	0.0152	0.0192	0.0103	0.0121	0.0322
T3+4	0.0224	0.0252	0.0447	0.0425	0.0907
PT2	0.6863	0.5087	0.6617	0.2952	0.0274
PT3	0.2367	0.1957	0.1444	0.0830	0.0335
PT4	0.1358	0.2567	0.1183	0.1531	0.0209
PSC1	0.5393	0.5110	0.4478	0.2146	0.1421
PSC2	0.0000	1.0264	0.5495	1.1083	0.3058
PLAB1	0.0000	0.0288	0.0215	0.0240	0.0000
PLAB2	0.0000	0.1100	0.1453	0.4239	0.0000
FWL	0.0053	0.0408	0.0039	0.0080	0.0196
FWW	0.0065	0.0278	0.0092	0.0122	0.0185
CUB A	0.0265	0.0512	0.0235	0.0432	0.0418
CUB B	0.0855	0.0732	0.0805	0.0717	0.1000
CI	0.0662	0.0831	0.0774	0.1209	0.1385
A4	0.0119	0.0284	0.0280	0.0389	0.0323
B4	0.0089	0.0276	0.0254	0.0141	0.0134
D7	0.0113	0.0312	0.0212	0.023	0.0142
E9	0.0310	0.0644	0.0428	0.0245	0.0298
G18	0.0193	0.0111	0.0112	0.0243	0.0177
J10	0.0273	0.0190	0.0237	0.0115	0.0288
J16	0.0109	0.0238	0.0168	0.0135	0.0178
K19	0.0096	0.0141	0.0127	0.0135	0.0225
L13	0.0208	0.0427	0.0382	0.0633	0.0534
N23	0.0160	0.0242	0.0283	0.0159	0.0159
O26	0.0226	0.0465	0.0403	0.0261	0.0542

Note: Except the samples from Shandong, the minimum values are shown in blue. The maximum values are shown in red.

Table S4. Predicted group membership information of stepwise discriminant analysis.

	Population	Maerkang	Danba	Batang	Derong	Linzhichayu	Linzhibomi	Diqingweixi	Diqingdeqin	Diqingxianggelila	Minhe	Xunhua	Tanchangxian	Lintan	Zhuoni	Minxian	Wenxian	Shandong
	Maerkang	7	0	0	0	0	0	1	0	0	0	0	0	0	0	0	0	0
	Danba	0	3	0	0	0	0	0	0	0	0	0	0	0	0	0	0	0
	Batang	0	0	4	0	0	0	0	2	0	0	0	0	0	0	0	0	0
	Derong	0	0	0	3	0	0	0	1	0	0	0	0	0	0	0	0	0
	Linzhichayu	0	0	0	0	5	0	0	0	0	0	0	0	0	0	0	0	0
	Linzhibomi	0	0	0	0	1	6	0	0	0	0	0	1	0	0	0	0	0
	Diqingweixi	0	0	0	1	0	0	7	0	3	1	0	2	0	1	0	1	0
	Diqingdeqin	0	0	1	0	0	0	1	3	0	0	0	0	0	0	0	1	0
count	Diqingxianggelila	0	0	0	0	0	0	1	1	3	0	0	0	0	0	0	2	0
	Minhe	0	0	0	0	0	0	0	0	0	3	0	0	0	0	0	0	0
	Xunhua	0	0	0	0	0	0	0	0	0	0	2	0	0	0	0	0	0
	Tanchangxian	0	0	0	0	0	0	0	0	1	0	0	2	0	0	0	1	0
	Lintan	0	0	0	0	0	0	0	0	0	0	0	0	2	0	1	0	0
	Zhuoni	1	0	0	0	0	0	1	0	0	2	0	0	0	1	0	0	1
	Minxian	0	0	0	0	0	0	0	0	1	2	0	0	1	1	4	0	0
	Wenxian	0	0	0	0	0	0	0	0	0	0	0	0	1	1	0	3	0
	Shandong	0	0	0	0	1	0	0	0	0	0	0	0	0	1	0	0	8
	Maerkang	87.5	0	0	0	0	0	12.5	0	0	0	0	0	0	0	0	0	0
	Danba	0	100	0	0	0	0	0	0	0	0	0	0	0	0	0	0	0
	Batang	0	0	66.7	0	0	0	0	33.3	0	0	0	0	0	0	0	0	0
	Derong	0	0	0	75	0	0	0	25	0	0	0	0	0	0	0	0	0
	Linzhichayu	0	0	0	0	100	0	0	0	0	0	0	0	0	0	0	0	0
	Linzhibomi	0	0	0	0	12.5	75	0	0	0	0	0	12.5	0	0	0	0	0
	Diqingweixi	0	0	0	6.3	0	0	43.8	0	18.8	6.3	0	12.5	0	6.3	0	6.3	0
	Diqingdeqin	0	0	16.7	0	0	0	16.7	50	0	0	0	0	0	0	0	16.7	0
%	Diqingxianggelila	0	0	0	0	0	0	14.3	14.3	42.9	0	0	0	0	0	0	28.6	0
	Minhe	0	0	0	0	0	0	0	0	0	100	0	0	0	0	0	0	0
	Xunhua	0	0	0	0	0	0	0	0	0	0	100	0	0	0	0	0	0
	Tanchangxian	0	0	0	0	0	0	0	0	25	0	0	50	0	0	0	25	0
	Lintan	0	0	0	0	0	0	0	0	0	0	0	0	66.7	0	33.3	0	0
	Zhuoni	16.7	0	0	0	0	0	16.7	0	0	33.3	0	0	0	16.7	0	0	16.7
	Minxian	0	0	0	0	0	0	0	0	11.1	22.2	0	0	11.1	11.1	44.4	0	0
	Wenxian	0	0	0	0	0	0	0	0	0	0	0	0	20	20	0	60	0
	Shandong	0	0	0	0	10	0	0	0	0	0	0	0	0	10	0	0	80

Table S5. Cross-verified predicted group membership information of stepwise discriminant analysis.

	Population	Maerkang	Danba	Batang	Derong	Linzhichayu	Linzhibomi	Diqingweixi	Diqingdeqin	Diqingxianggelila	Minhe	Xunhua	Tanchangxian	Lintan	Zhuoni	Minxian	Wenxian	Shandong
	Maerkang	5	0	0	1	0	0	1	0	0	0	0	0	0	0	1	0	0
	Danba	0	3	0	0	0	0	0	0	0	0	0	0	0	0	0	0	0
	Batang	0	0	4	1	0	0	0	1	0	0	0	0	0	0	0	0	0
	Derong	0	0	0	3	0	0	0	1	0	0	0	0	0	0	0	0	0
	Linzhichayu	0	0	0	0	5	0	0	0	0	0	0	0	0	0	0	0	0
	Linzhibomi	0	0	0	0	1	4	0	0	0	0	0	2	0	0	0	0	1
	Diqingweixi	0	0	0	2	0	0	5	0	3	1	0	2	0	1	1	1	0
	Diqingdeqin	0	0	1	1	0	0	1	2	0	0	0	0	0	0	0	1	0
count	Diqingxianggelila	0	1	0	0	0	0	2	1	1	0	0	0	0	0	0	2	0
	Minhe	0	0	0	0	0	1	0	0	0	1	0	0	1	0	0	0	0
	Xunhua	0	0	0	0	0	0	0	0	0	1	1	0	0	0	0	0	0
	Tanchangxian	0	0	0	0	0	0	2	0	1	0	0	0	0	0	0	1	0
	Lintan	0	0	0	0	0	0	0	0	1	0	0	0	0	0	1	1	0
	Zhuoni	1	0	0	0	0	0	1	0	0	2	0	0	0	0	1	0	1
	Minxian	0	0	0	0	0	0	0	0	1	2	0	0	2	1	3	0	0
	Wenxian	0	0	0	0	0	0	0	0	0	0	0	0	1	1	0	3	0
	Shandong	0	0	0	0	1	0	0	0	0	0	0	0	0	2	0	0	7
	Maerkang	62.5	0	0	12.5	0	0	12.5	0	0	0	0	0	0	0	12.5	0	0
	Danba	0	100	0	0	0	0	0	0	0	0	0	0	0	0	0	0	0
	Batang	0	0	66.7	16.7	0	0	0	16.7	0	0	0	0	0	0	0	0	0
	Derong	0	0	0	75	0	0	0	25	0	0	0	0	0	0	0	0	0
	Linzhichayu	0	0	0	0	100	0	0	0	0	0	0	0	0	0	0	0	0
	Linzhibomi	0	0	0	0	12.5	50	0	0	0	0	0	25	0	0	0	0	12.5
	Diqingweixi	0	0	0	12.5	0	0	31.3	0	18.8	6.3	0	12.5	0	6.3	6.3	6.3	0
	Diqingdeqin	0	0	16.7	16.7	0	0	16.7	33.3	0	0	0	0	0	0	0	16.7	0
%	Diqingxianggelila	0	14.3	0	0	0	0	28.6	14.3	14.3	0	0	0	0	0	0	28.6	0
	Minhe	0	0	0	0	0	33.3	0	0	0	33.3	0	0	33.3	0	0	0	0
	Xunhua	0	0	0	0	0	0	0	0	0	50	50	0	0	0	0	0	0
	Tanchangxian	0	0	0	0	0	0	50	0	25	0	0	0	0	0	0	25	0
	Lintan	0	0	0	0	0	0	0	0	33.3	0	0	0	0	0	33.3	33.3	0
	Zhuoni	16.7	0	0	0	0	0	16.7	0	0	33.3	0	0	0	0	16.7	0	16.7
	Minxian	0	0	0	0	0	0	0	0	11.1	22.2	0	0	22.2	11.1	33.3	0	0
	Wenxian	0	0	0	0	0	0	0	0	0	0	0	0	20	20	0	60	0
	Shandong	0	0	0	0	10	0	0	0	0	0	0	0	0	20	0	0	70
